# The potential of task-shifting in scaling up services for prevention of mother-to-child transmission of HIV: a time and motion study in Dar es Salaam, Tanzania

**DOI:** 10.1186/s12960-017-0207-2

**Published:** 2017-05-26

**Authors:** Helga Naburi, Anna Mia Ekström, Phares Mujinja, Charles Kilewo, Karim Manji, Gunnel Biberfeld, David Sando, Guerino Chalamila, Till Bärnighausen

**Affiliations:** 1 Department of Pediatrics and Child Health, Muhimbili University of Health and Allied Sciences (MUHAS), Dar es Salaam, Tanzania; 2 Department of Public Health Sciences, Global Health (IHCAR), Karolinska Institutet, Stockholm, Sweden; 3 Department of Infectious Diseases, Karolinska University Hospital, Huddinge, Stockholm, Sweden; 4 School of Public Health, Muhimbili University of Health and Allied Sciences (MUHAS), Dar es Salaam, Tanzania; 5 Department of Obstetrics and Gynaecology, Muhimbili University of Health and Allied Sciences (MUHAS), Dar es Salaam, Tanzania; 6 Department of Global Health and Population, Harvard T.H. Chan School of Public Health, Boston, MA United States of America; 7 Management and Development for Health (MDH), Dar es Salaam, Tanzania; 8 Africa Health Research Institute (AHRI), Somkhele, South Africa; 9 Institute for Public Health, University of Heidelberg, Heidelberg, Germany

**Keywords:** Task-shifting, Nurses, Community health workers, Cost, Prevention of mother-to-child transmission, Option B+, Time and motion, Dar es Salaam

## Abstract

**Background:**

In many African countries, prevention of mother-to-child transmission of HIV (PMTCT) services are predominantly delivered by nurses. Although task-shifting is not yet well established, community health workers (CHWs) are often informally used as part of PMTCT delivery. According to the 2008 World Health Organization (WHO) Task-shifting Guidelines, many PMTCT tasks can be shifted from nurses to CHWs.

**Methods:**

The aim of this time and motion study in Dar es Salaam, Tanzania, was to estimate the potential of task-shifting in PMTCT service delivery to reduce nurses’ workload and health system costs. The time used by nurses to accomplish PMTCT activities during antenatal care (ANC) and postnatal care (PNC) visits was measured. These data were then used to estimate the costs that could be saved by shifting tasks from nurses to CHWs in the Tanzanian public-sector health system.

**Results:**

A total of 1121 PMTCT-related tasks carried out by nurses involving 179 patients at ANC and PNC visits were observed at 26 health facilities. The average time of the first ANC visit was the longest, 54 (95% confidence interval (CI) 42–65) min, followed by the first PNC visit which took 29 (95% CI 26–32) minutes on average. ANC and PNC follow-up visits were substantially shorter, 15 (95% CI 14–17) and 13 (95% CI 11–16) minutes, respectively. During both the first and the follow-up ANC visits, 94% of nurses’ time could be shifted to CHWs, while 84% spent on the first PNC visit and 100% of the time spent on the follow-up PNC visit could be task-shifted. Depending on CHW salary estimates, the cost savings due to task-shifting in PMTCT ranged from US$ 1.3 to 2.0 (first ANC visit), US$ 0.4 to 0.6 (ANC follow-up visit), US$ 0.7 to 1.0 (first PNC visit), and US$ 0.4 to 0.5 (PNC follow-up visit).

**Conclusions:**

Nurses working in PMTCT spend large proportions of their time on tasks that could be shifted to CHWs. Such task-shifting could allow nurses to spend more time on specialized PMTCT tasks and can substantially reduce the average cost per PMTCT patient.

## Background

The number of people living with HIV (PLHIV) in Tanzania in 2015 was estimated to be 1 400 000, equating to approximately 4.7 in every 100 people, and 56% of those living with HIV are women aged 15 years and above [1]. Mother-to-child transmission (MTCT) of HIV rates in Tanzania were 8% in 2015 [2]. These MTCT rates put Tanzania in the middle range of PMTCT success in sub-Saharan Africa. Some countries in the region, such as South Africa and Swaziland, have achieved MTCT rates below 5%. Others, such as Angola and Nigeria have MTCT rates above 20% [3].

Between October 2013 and April 2014, Tanzania started to implement the WHO’s recommended strategy for the prevention of mother-to-child transmission of HIV (PMTCT), referred to as Option B+ [4]. It entails providing lifelong triple antiretroviral therapy (ART) to all pregnant women living with HIV irrespective of CD4 cell count [5]. At full scale-up, Option B+ is expected to increase the number of women starting lifelong ART by 60% [6], requiring a rapid expansion of the current health workforce to meet this demand. In general, staff shortages are among the primary constraints to the provision of universal access to PMTCT and ART [7–9].

In Tanzania, PMTCT services are integrated into antenatal care (ANC). Nearly all pregnant women in the country (98%) make at least one visit to an ANC facility, and 90% of them test for HIV [4]. The integration of PMTCT into ANC decreases patient time spent at the health facility and leads to a closer provider-patient relationship, but it also increases the average amount of time ANC nurses spend with their clients [10].

Despite an increase in the health workforce over the last 6–7 years [11], the population density of health care workers in Tanzania (5 per 10 000 people) is far below the WHO recommended minimum density (23 health workers per 10 000 people) [12]. Tanzania currently averages 0.26 medical doctors, 0.40 assistant medical officers, 0.56 nurse officers, and 4.2 enrolled nurses and nurse midwives per 10 000 population [13, 14]. ANC and PMTCT services are typically provided by nurse officers, enrolled nurses, or nurse midwives [14]. In addition to the limited human resources for health, shortage of supplies including equipment and medication are other constraints that reduce the quality and efficiency of PMTCT services [15].

In response to the human resources crisis in areas with high HIV burden [16, 17], the WHO in collaboration with UNAIDS (Joint United Nations Programme on HIV/AIDS) and PEPFAR (President’s Emergency Plan for AIDS Relief) published guidelines in 2008 for the implementation of task-shifting as a part of the solution to rapidly increase access to health services for PLHIV [18]. According to WHO, community health workers (CHWs) could potentially deliver a wide range of services including PMTCT, under supportive supervision, freeing up time for more qualified providers to deliver more specialized care [17, 18].

CHWs are often proposed to perform the bulk of work in primary health care facilities [19–26]. In many countries, models of care have been adopted that use CHWs for the delivery of primary-care services, in particular in HIV, maternal, and child health care [9, 21, 27–29]. In Tanzania, lower cadre of health workers such as the so-called medical attendants are already doing tasks of nursing officers, and many clinical officers perform medical officers’ tasks, but task-shifting to CHWs has not yet been formally established [30].

The CHW model was first introduced in Tanzania following the 1978 Alma Ata Declaration [31]. This declaration underlined the importance of primary health care (PHC) as the means for providing equitable and affordable health care service to all, also recognizing the importance of using trained CHWs for rapid expansion of the health workforce. Following the global economic and political crisis in the 1980s and 1990s, PHC and CHW programs were left uncoordinated in many countries [32].

Currently, about 41 000 CHWs are employed across Tanzania by a network of development partners and non-governmental organizations. Only about one in four of these CHWs have at least a form-four-level education (4 years of secondary education), which is required for the eligibility to be on the government payroll. Thus, the majority of CHWs are employed on a voluntary basis [31]. The training of CHWs in Tanzania ranges from 1 week to 9 months, and the duties and tasks of CHWs differ widely depending on the project and the sponsor. However, about three quarters of CHWs in the country have knowledge on health promotion and one out of two has been trained in HIV-related tasks, such as HIV testing and counselling [31].

The evidence on the potential contributions of CHWs in reducing child and maternal mortality has driven a renewed interest in CHW programs in many low- and middle-income countries [33]. Thus, recognizing the current human resources shortage and considering the future need, Tanzania plans to systematically involve trained and supervised CHWs into task-shifting arrangement to improve access to health care. The National CHW task force has been established to advise the Ministry of Health, Community Development, Gender, Elderly and Children (MoHCDGEC) on the matters related to the CHW policy, strategies, and guidelines, as well the scope of the CHWs’ training, task, and remunerations [34].

To inform this policy agenda, this time and motion study measured the average time nurses spent on delivering the different tasks that make up PMTCT both in ANC and in postnatal care (PNC). This data was then used to estimate the reductions in costs and in nurses’ workload that could be achieved by systematically shifting tasks from nurses to CHWs in health care facilities providing PMTCT in the public-sector health system in Dar es Salaam, Tanzania.

## Methods

### Study setting

This study was conducted in health facilities sampled from all public-sector PMTCT facilities in two of the three districts of Dar es Salaam, which together hosts about 70% of the 4.4 million people residing in the city [35]. During the study, Tanzania was in the process of phasing out Option A (zidovudine during pregnancy coupled with infant nevirapine during breastfeeding for women without advanced HIV disease) and adopting Option B+. PMTCT services in Tanzania are offered according to the National guidelines, adopted from WHO, describing all activities that should be carried out at each antenatal and postnatal visit [4].

### Data collection

Between March and April 2014, a time and motion study was carried out to establish PMTCT task times. The observers followed one nurse each during working hours to record the time she spent on various PMTCT-related activities. Three research assistants (recently graduated Tanzanian doctors) were recruited to serve as observers. They received practical training for 2 weeks on the research protocol, the data collection tools, and time and motion study methods. All study instruments were piloted and revised after the pilot study to ensure reliability, content validity, and quality of the data collection tools before initiating the study. The first author (HN), a medical doctor experienced in quantitative surveys, supervised the training and data collection process to ensure the quality and consistency of the data collected.

To minimize potential Hawthorne effects (whereby nurses could modify their work performance because they were being observed) [36], the research assistants explained succinctly that the aim of the study was to estimate the time routinely spent on PMTCT activities, that no personal identifiers would be collected and that the data would only be used for scientific purposes and not for work performance evaluation.

Written informed consent was obtained from both the health worker and the patient before observation, and no incentive was given to the study participants. All data were anonymously collected without any identifying information on the health workers or patients. Permission to work in the clinics was obtained from both the district and health facility authorities in charge. The Research and Ethics Committee of Muhimbili University of Health and Allied Sciences (MUHAS) approved the study.

### Sampling

The study population comprised of all nurses working in PMTCT services in public-sector ANC facilities in the Kinondoni and Ilala districts of Dar es Salaam, Tanzania. The study was conducted only in public-sector health care facilities, because health workforce shortages are much more severe in the public than in the private sector. Moreover, the vast majority of the population utilizes the public sector for most of their health care needs, while only a comparatively small population, typically those with high income and high educational attainment, utilizes private care. There were 58 public-sector facilities that offered integrated ANC and PMTCT services in these two districts. From these facilities, all those (*N* = 26) with a record of registering at least one new HIV-positive pregnant woman per month were selected. Each of the three external observers employed for this study was randomly assigned a facility each day over the observation period. On each of the sampled facility days, the observer visited the facilities and randomly selected nurse-patient interactions for the time and motion observation. The nurse-patient interactions were observed in real time, using a stopwatch and a time sheet to measure and record the time spent in minutes for each task performed by the nurses. The observation started when a nurse-patient contact was initiated and ended when the nurse had completed a task. The nurse-patient interactions were observed during four different types of PMTCT visits: the first ANC PMTCT visit, ANC PMTCT follow-up visits, the first postnatal care (PNC) PMTCT visit, and the subsequent PNC PMTCT follow-up visits.

### This time and motion study in context

Frederick Taylor and Frank and Lilian Gilbreth developed the time and motion method in the early 20th century, to improve the efficiency of industrial processes [37, 38]. In 1914, the Gilbreths applied the method for the first time to study task times in health care facilities [39]. Following a recent classification by Lopetegui et al. [40], the particular variant of a time and motion study that was carried out here was an “external-observer” study, because external observers (recent medical graduates from Tanzania) were employed to carry out the task time observation. It was further a “continuous observation” study, because the external observer maintained attention on one nurse-patient interaction, continuously recording the time taken to all tasks related to one PMTCT visit. Finally, it was a “workflow time study” because the duration of the PMTCT-related tasks recorded by the external observers occurred at unpredictable times during the day and in unpredictable patterns and sequences.

### Data analysis

Data from the time sheets were entered into an Excel spreadsheet (Microsoft Corp., Redmond, WA, USA), cleaned, coded, and imported into Stata software for analysis (Stata Statistical Software, Release 14, Stata Corp 2014). The tasks performed during the different types of PMTCT visits (ANC versus PNC visits; first versus follow-up visits) were mapped in relation to the tasks described in the WHO document *Task-Shifting – Global Recommendations and Guidelines* [18]. The average time spent on each of the PMTCT activities was analyzed. This data was used to calculate the total time that could be task-shifted if CHWs executed such tasks that according to the WHO can be carried out by CHWs. Unit costs were calculated by converting monthly salaries to salaries per minute work time. The unit cost was multiplied by the time spent in each task to obtain cost per task.

The average cost of a visit before task-shifting was calculated based on the weighted average salary for each of the four types of visits observed. These weighted averages were calculated, because different nurse cadres were observed carrying out different proportions of these visit times: nurse officers (who earn a salary of US$ 495/month) and enrolled nurses and nurse midwives (who earn a salary of US$ 438/month). To calculate the potential cost savings due to shifting of tasks from these nurse cadres to CHWs, three different CHW salary estimates were used: the minimum salary for a formally trained Tanzanian health worker equivalent to a CHW level in the public-sector health system (US$ 172 per month) [41], the remunerations paid to lay health workers in an empirical study (US$ 30 per month) [42, 43], and the upper bound of the remuneration range for CHWs in Tanzania, which was recommended based on a normative analysis (US$ 150) [44]. All costs were converted from Tanzanian shillings (TZS) to US dollars (US$), using the average exchange rate for the month of December 2015 (1 USD = 2154 TZS). The monthly salary estimates were converted to minute salary estimates, assuming 22 working days per month and 8 working hours per day.

## Results

A total of 1121 PMTCT-related tasks involving 179 patient-nurse interactions were observed. The total observation time was 4551 min or approximately 76 h. About 57% of the observations were from lower-level health facilities (so-called dispensaries) and 43% were from health centers and hospitals (Table 1). The distribution of total visit times between nurse officers versus enrolled nurses or nurse midwives was 19 versus 81% for the first ANC PMTCT visit, 41 versus 59% for the ANC PMTCT follow-up visit, 53 versus 47% for the first PNC PMTCT visit, and 50 versus 50% for the PNC PMTCT follow-up visit.

**Table 1:** Baseline characteristics of 179 nurses observed in 26 health facilities in Kinondoni and Ilala districts, Dar es Salaam, Tanzania

Variable	Proportion of observations in %
District	
Kinondoni	51
Ilala	49
Health facility level	
Dispensary	57
Health centers and hospitals	43
Nurse cadre	
Nurse officers	36
Nurse midwife and enrolled nurse	64

Training times vary between different nurse cadres: nurse officers train for 3–4 years while enrolled nurses and nurse midwives train for 2–3 years

The first ANC PMTCT visit was the longest, which took an average time length of 54 (95% CI 42–65) minutes. The largest proportion of time during this visit was spent on maternal HIV testing; with pre- and posttest counselling, the entire HIV testing and counselling process accounted for 44% of visit time. During this visit, 22% of the time was spent on documentation, including filling out the PMTCT registers and taking map cues (detailed descriptions of the location of a patient’s residence).

The ANC follow-up visits were substantially shorter with an average time length of 15 (95% CI 14–17) minutes. The largest proportion of time (73%) during this visit was spent on infant feeding counselling, filling registers, and checking for ART adherence (Table 2).

**Table 2:** Average time spent per patient on different PMTCT tasks

Task	Observations (*N*)	% time	Mean task time (minutes)	95% CI
First antenatal PMTCT visit				
Pretest counselling	36	16.2	8.7	6.1–11.3
HIV testing (rapid test)	36	12.1	6.5	4.0–9.0
Filling lab forms	16	2.6	3.1	2.6–3.7
Giving HIV results	36	5.8	3.1	2.5–3.6
Posttest counselling	36	9.5	5.1	3.9–6.3
Filling log book	36	4.7	2.5	2.1–3.0
Filling PMTCT care register	34	5.5	3.1	2.6–3.7
Map cue	27	11.5	8.2	6.3–10.1
ART adherence counselling	25	9.6	7.4	4.4–10.5
Filling CTC no. 1^a^ and no. 2^b^	25	7.0	5.4	4.0–6.9
HIV disease staging (WHO)^c^	26	3.1	2.3	1.9–2.7
Taking blood sample for CD4 count testing^c^	16	2.6	3.1	2.2–3.9
Dispensing antiretroviral drugs	26	4.6	3.4	2.5–4.4
Infant feeding counselling	25	4.9	3.8	3.2–4.4
Filling referral forms	3	0.3	2.0	2.0–2.0
Total	36	100	53.5	41.8–65.2
Antenatal PMTCT follow-up visit				
Filling PMTCT care register	59	24.4	4.3	3.7–4.9
Checking for ART adherence	62	23.8	4.0	3.0–4.9
HIV disease staging (WHO)^c^	24	6.2	2.7	2.3–3.0
Infant feeding counselling	47	25.7	5.7	5.0–6.4
Antiretroviral drug refill	61	19.9	3.4	2.8–3.9
Total	69	100	15.0	13.5–16.6

*CI* confidence interval, *ART* antiretroviral treatment

^a^Care and treatment cards (CTCs) are used in Tanzania for all registered patients living with HIV and contains a unique patient identification number and key information on patient management recorded during each visit

^b^CTC1 is an identification card kept by the patient; CTC2 is a record form kept at the clinic

^c^Tasks that can cannot be shifted from nurses to CHWs, according to WHO task-shifting guidelines [18]

The first PNC PMTCT visit was the second longest visit, with an average time length of 29 (95% CI 26–32) minutes. During this visit, infant HIV testing and counselling accounted for the largest proportion of time (34%). The PNC follow-up visits were substantially shorter with an average time length of 13 (95% CI 11–16) minutes, and the largest proportion of time (40%) was spent on filling out the registers (Tables 3).

**Table 3:** Average time spent in different tasks per patient per visit during postnatal PMTCT visits

Task	Observations (*N*)	% time	Mean task time (minutes)	95% CI
First postnatal PMTCT visit				
Assessing infant adherence to prophylaxis^a^	39	8.2	2.4	2.0–2.8
Assessing adherence to ART/maternal prophylaxis^a^	39	9.2	2.7	2.0–3.4
Assessing infant feeding	39	8.9	2.6	2.0–3.1
Pretest counselling	40	18.6	5.3	4.2–6.3
Filling mother infant register	38	11.6	3.5	2.9–4.1
Filling lab request forms	39	8.5	2.5	2.1–2.8
Filling log book	37	8.1	2.5	2.1–3.0
Updating Map cue^b^	5	2.5	5.6	3.7–7.5
Taking a dried blood spot (DBS) sample^c^	39	15.7	4.6	4.0–5.1
Dispensing cotrimoxazole^c^	40	8.8	2.5	2.5–3.2
Total	40	100	28.9	26.2–31.6
Postnatal PMTCT follow-up visit				
Filling mother infant follow-up register	28	40.2	6.3	5.0–7.5
Assessing infant feeding	23	25.7	4.9	4.0–5.8
Assessing infant adherence to prophylaxis^a^	27	12.3	2.0	1.5–2.4
Assessing adherence to maternal prophylaxis^a^	32	21.9	3.0	2.3–3.7
Total	34	100	13.1	10.8–15.5

*CI* confidence interval, *ART* antiretroviral treatment

^a^Adherence to maternal and infant prophylaxis was assessed using verbal reporting

^b^The map cue taken during the first ANC visit is updated during the first PNC visit

^c^Tasks that cannot be shifted from nurses to CHWs, according to WHO task-shifting guidelines [18]

**Fig. 1:**
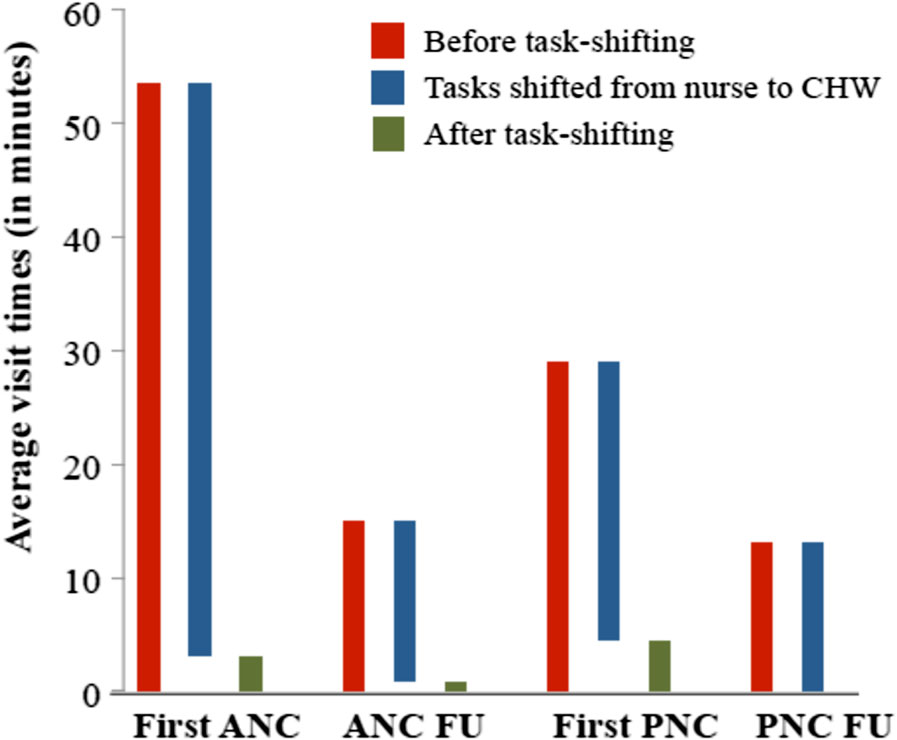
Task-shifting from nurses to community health workers. *CHW* community health worker, *First ANC* first antenatal care PMTCT visit, *ANC FU* antenatal care PMTCT follow-up visit, *First PNC* first postnatal care PMTCT visit, *PNC FU* postnatal care follow-up visit

Tables 2 and 3 show how often each task was observed, the average task time, and the proportion of total visit time that on average was spent on each task. Following the WHO guideline recommendations on which tasks that could be shifted from nurses to CHWs [18], it was found that the vast majority of the total visit time could be task-shifted: 94% during the first ANC PMTCT visit, 94% during the ANC PMTCT follow-up visit, 84% during the first PNC PMTCT visit, and 100% during the PNC PMTCT follow-up visit (Fig. 1).

Assuming that all tasks, which according to the WHO could be shifted from nurses to CHWs, were indeed task-shifted, the average cost per visit could be substantially reduced. Depending on the salary estimate for a CHW, the resulting per-visit cost savings would be between US$ 1.3 and 2.0 (for the first ANC PMTCT visit), US$ 0.4 and 0.6 (ANC PMTCT follow-up visit), US$ 0.7 and 1.0 (first PNC PMTCT visit), and US$ 0.4 and 0.5 (PNC PMTCT follow-up visit) (Table 4).

**Table 4:** Cost in US$, per PMTCT visit

Visits	Observations	Mean cost before task-shifting^a^	Mean cost after task-shifting^b^	Mean cost after task-shifting^c^	Mean cost after task-shifting^d^
First ANC visit	36	2.27	0.96	0.85	0.28
ANC follow-up visit	69	0.65	0.27	0.24	0.08
First PNC visit	40	1.28	0.60	0.55	0.27
PNC follow-up visit	34	0.58	0.21	0.19	0.04

Means costs in US$

*ANC* antenatal care, *PNC* postnatal care

^a^Calculated based on the weighted average salary of the publically employed nurse (3 observed cadres in Table 1) in Tanzania (nurse officer US$ 495, enrolled nurse and nurse midwife US$ 437)

^b^Calculated based on the minimum wage for a formally trained health worker in the public-sector health system in Tanzania (US$ 172 per month) [41]

^c^Calculated based on community health worker salaries proposed in a previous study for Tanzania (US$ 150 per month) [44]

^d^Calculated based on remuneration paid to lay health workers in another study (US$ 30 per month) [42]

## Discussion

This time and motion study measured the time spent by nurses to perform different PMTCT-related tasks in Dar es Salaam, Tanzania. Using this data, we estimated the potential effects of task-shifting PMTCT services from nurses to CHWs on health care costs and nurses’ workload. The majority of the time that nurses currently spend on PMTCT-related tasks could be shifted to CHWs, saving substantial amounts of money per patient visit. The saved resources could be re-allocated to other health care activities to increase patient access to quality health services. For instance, nurses could use the time freed up by task-shifting to provide the complex and specialized services that CHWs cannot carry out, such as assessing PMTCT patients for co-morbidities. The time and the money freed up by task-shifting could also be used to support the scaling up of the PMTCT Option B+ and family planning services to benefit the around 95 000 women living with HIV who become pregnant every year in Tanzania [2].

It has been estimated that the number of women who start lifelong ART in pregnancy would increase substantially as PMTCT Option B+ is rolled out globally [45]. The goal of Option B+ is to ensure universal access to ART for all pregnant and breastfeeding women living with HIV. However, the overall shortage of health workers [12] and the unequal distribution of health workers across rural and urban facilities [13] may result in unequal scale-up of this intervention across population and geographic groups. Task-shifting could increase the technical efficiency of PMTCT service delivery [23, 27, 28], and thus, more PMTCT services can be provided for given resources. Whether task-shifting does indeed increase the cost-effectiveness of PMTCT service delivery or not will depend critically on the effectiveness side of the equation; it is plausible that at the same time as costs of service provision decreases, so does the quality of care and effectiveness of PMTCT. Real-life scale-up of CHW-supported PMTCT delivery models needs to be accompanied by evaluations assessing the quality of care and effectiveness of these models compared to delivery models without CHW.

CHW-supported PMTCT delivery models have the potential to substantially increase access to PMTCT, because more services can be provided for given budgets when CHWs take over a range of tasks that have so far been delivered by nurses. The resulting increase in access to PMTCT could also affect the distribution of health outcomes and have equity implications. For instance, under a utilitarian conception of equity, an increase in the provision of needed PMTCT services implies an improvement in equity of health outcomes. Similarly, under many other conceptions of equity, it is likely that task-shifting, while keeping the resources available for PMTCT constant, will improve equity in health outcomes [46]. In many settings, the most vulnerable are least able to successfully negotiate access to scarce health care services including PMTCT [47]. Thus, as overall access increases, the most vulnerable will be increasingly able to utilize needed PMTCT services. This situation will imply an equity improvement under those conceptions of equity that prioritize the most over the least vulnerable. In addition, the use of CHW in the delivery of PMTCT would also be a comparatively fast approach to increasing the health system capacity for PMTCT, because the time it takes to train a CHW is manifold shorter than the time it takes to train a nurse.

Findings from this study show that activities that require direct communication and interactions with clients, such as counselling sessions and adherence assessments, on average took more time than other activities. This finding is plausible and is also in line with previous research [48, 49]. Successful communication and interaction takes time because health workers need to explain and reinforce the main PMTCT messages, and health workers need to allow clients adequate time to consider and query the information they have received [50]. Since it is unlikely that communication task times could be substantially reduced without an accompanying reduction in quality, theoretically, it is indeed task-shifting (rather than task time reduction) that holds promise to increase technical efficiency in the delivery of PMTCT services in Tanzania and other countries where the pool of health workers cannot be rapidly expanded [21]. While task-shifting from nurses to CHWs can free up nurse capacity and save costs, in countries with low nurse-to-population ratios, task-shifting initiatives should not lead to a reduced effort to train, recruit, and retain more nurses.

In addition to counselling, WHO recommends task-shifting to CHWs for activities involving documentation, such as filling out PMTCT registers and care and treatment cards [18]. Documentation ranked second in terms of time spent across all categories of tasks, incurring a significant part of the costs associated with PMTCT services. A particular documentation task that took a comparatively long time to complete was the map cue recording. Map cues are detailed descriptions of the location of a patient’s residence and how to reach the patient from the PMTCT clinic. Map cues allow health workers to follow up patients in their homes, especially those who fail to return for scheduled clinic visits. Since proper documentation and record keeping is an essential part of patient care, shifting documentation from nurses to trained CHWs could contribute to reducing the workload for nurses and increase their focus on more specialized and complex tasks, such as clinical HIV staging and assessment of pregnancy-related risks. Such re-allocation of tasks may also increase the quality of care, because nurses’ time and attention is not wasted on routine tasks but focused on activities that require a higher level of training and skills [51]. This will also improve patient satisfaction, because the quality of care and the time spent in direct patient services, including counselling, is an important determinant of patient satisfaction [52].

In the current empirical study, nurses spent a fair proportion of their time on blood tests, including HIV testing, which WHO suggests can be successfully carried out by CHWs. However, nurses also spent substantial amounts of time on tasks that WHO does not recommend to be shifted to CHWs, such as drawing venous blood for CD4 counts and taking dried blood spots for DNA PCR. These task-shifting recommendations imply that, in the context of PMTCT Option B+, all HIV testing and counselling of pregnant women at the first ANC visit could be task-shifted to CHWs, allowing nurses to perform the more specialized blood tests and the associated complex patient assessments.

WHO recommends that counselling tasks can be shifted to CHWs, but there may be some concerns that the quality of counselling will suffer if it is offered by CHWs, because CHWs may have received less counselling training than nurses or patients may not value nurse and CHW counselling interactions equally. However, the empirical evidence suggests that well-trained and supervised CHWs can indeed be successful in delivering good quality HIV counselling and testing [24, 53].

In general, even relatively complex tasks can likely be successfully executed by CHWs given sufficient training and experience [54]. Trained and supervised CHWs may be able to perform more tasks than those recommended for task-shifting by the WHO guidelines, for instance, the provision of injectable contraceptives can probably be safely and effectively carried out by CHWs [54]. Hence, another important focus for task-shifting to CHWs may be increasing access to family planning and contraception services which would translate into reduced ANC and PMTCT workload as well as health system costs in the future.

Option B+ PMTCT is expected to substantially increase the number of women in need of ART, adding pressures on African health systems [45], which are already facing severe health worker shortages [11, 13, 55]. Given the existing constraints in health worker training capacity in sub-Saharan Africa, implementing programs to substantially and rapidly expand the pool of nurses to provide the additional care needed under PMTCT Option B+ may not be feasible for low-income countries like Tanzania [17]. In addition to challenges with training and expanding the workforce, switching to other jobs is also a key factor contributing to the overall staff shortage in public health facilities in Tanzania. The main reasons for job change in low-income countries are the excessively high workload in relation to low salary [56]. In such a situation, task-shifting could become an important part of a feasible and cost-effective solution to temporarily fill the gap of human resources for health [17, 30] and to maintain patient access to health care. However, the critical determinants of the success of CHW and task-shifting programs include CHW remuneration, recognition of performance, and involvement in decisions affecting daily work routines [18, 24, 30]. Thus, the overall success and sustainability of task-shifting to CHWs in Tanzanian health system will depend on the work conditions, performance management, and continued training.

The analyses in this paper assumed that CHWs would work in health care facilities side by side with nurses. Similar delivery models of nurses and lower-skilled health workers working side by side are already practiced in many HIV treatment programs in Eastern and Southern Africa [23, 27, 28, 53] and currently under consideration in Tanzania. However, it is of course also possible that a CHW would carry out some of the PMTCT tasks in other settings, e.g., in patients’ homes. Such distribution of tasks across different settings is constrained by the fact that some tasks are necessarily or better delivered in an uninterrupted workflow. Because not all PMTCT-related tasks can be shifted to CHWs and nurses are unlikely to deliver PMTCT tasks outside the clinic setting, home-based delivery of PMTCT may be difficult given the current guidelines and delivery models but also due to stigma and unwanted disclosure of HIV status. Thus, assigning CHWs to work as health attendants in the PMTCT clinics as a primary task rather than an additional task could result in more efficiency gain and cost saving while ensuring the delivery of quality care.

In this analysis, the assumption that the 2008 WHO task-shifting guidelines are valid for Tanzania was made. As described in the guidelines publication, the process followed by the WHO in determining the task-shifting recommendations was rigorous:

The recommendations and guidelines on task-shifting were developed based on the existing evidence that is available in both published literature and in “grey” literature such as policy documents and reports. These sources were complemented by evidence gathered through specifically commissioned studies in seven selected countries that had different degrees of experience in task-shifting. The evidence gathering was informed and guided by a wide range of experts and stakeholders. A panel of experts reviewed the evidence over a period of one year at a total of nine international consultations and agreed on the final text [18].

In addition to the overall rigorous process, the technical experts and stakeholders who contributed to the guidelines included two experts from Tanzania. However, Tanzania-specific evidence on the performance of task-shifting was lacking when the recommendations were determined. It is thus possible that some of the tasks deemed safe to shift from nurses to CHWs should not be shifted in Tanzania. In this case, the cost savings reported will be an overestimate of actual cost savings. As Tanzania and other countries increasingly shift PMTCT tasks from nurses to CHWs, evaluation studies should determine whether these initiatives have unintended consequences, such as reductions in quality of care in PMTCT. As part of the dissemination strategy by the team of investigators who carried out this study, the study findings will be discussed with government and non-government stakeholders in Tanzania.

This study quantifies the potential savings and efficiency gains that can be achieved through task-shifting in PMTCT from a nurse to a CHW, which can also be translated to other cadres. The capacity gains that could result from task-shifting are large and can likely contribute to ensuring that Tanzania achieves important PMTCT-related health targets, such as eliminating new HIV infections among children and keeping mothers alive, in addition to the UNAIDS “fast track” goal of ending the HIV epidemic by 2030.

As CHWs can substantially decrease the workload of health care workers [53], it is plausible that given the current health workforce shortages, nurses may accept that some of their duties are task-shifted to CHWs. Indeed, it might enhance nurses’ job satisfaction and professional identity if they increasingly performed tasks that highly require their expertise. However, while some level of task-shifting in Tanzania is already informally practiced [30, 57], nurses may initially refuse formal task-shifting initiatives, for instance, because they are not prepared for the additional supervisory responsibilities that come with task-shifting or because they fear losing professional status. Thus, the task-shifting initiatives should be carefully planned and discussed with nurses and other health care professionals. Additionally, it will be important that major task-shifting initiatives are rigorously assessed in their performance and their causal impact on health outcomes, to ensure that lack of success and unintended consequences are identified and remedial action can be taken.

Limitations to this study include the calculation of cost savings, since the assumption was made that CHWs would need the same amount of time as existing trained and experienced nurses to deliver a particular PMTCT service. This assumption may not be correct; however, given the size of the cost savings per PMTCT visit, it is very likely that major cost savings can be achieved through task-shifting, even if CHWs needed somewhat more time than nurses to execute particular tasks. Furthermore, it has been shown across a number of settings that health cadres with comparatively short training become very efficient in executing particular tasks and delivering a narrow set of services with time and repetition [54, 58]. Thus, it is plausible that CHWs initially will take a bit longer to fulfill certain tasks, but CHWs are much cheaper and much easier to train than nurses, so if they take a bit longer, the savings in terms of scarce nurse time and money will still be large [57].

An explanation to why the cost (and time) savings may be less than calculated is the fact that CHWs are likely to require some level of supervision by nurses. However, it is clear that nurses could substantially leverage their own time when supervising task-shifting to CHWs, ensuring that the task-shifting will lead to major cost savings, even if they may be slightly lower than the results in this study suggest. Moreover, many of the tasks that can be shifted to CHWs are fairly simple, and CHWs may, with time, only need minimal supervision in carrying them out [54].

Another potential limitation of this study is that observation bias could have been introduced through the approach used in this study of directly and openly observing provider-patient interactions. However, the time observed to complete specific tasks, such as pretest counselling, was consistent with results from other studies in Tanzania that used different methods to measure task times [49, 59]. Moreover, if observation bias is present in a study, it has commonly been found that it decreases over time as the process of observation becomes increasingly “routine.” In this study, average task times did not change over the observation period, indicating that observation bias was unlikely to have played a major role in this study.

## Conclusions

Nurses working in PMTCT spend large proportions of their time on tasks that could be shifted to CHW. Such task-shifting could allow nurses to spend more time on specialized PMTCT tasks and might substantially reduce the average cost per PMTCT patient. Thus, task-shifting appears to be one important option that could substantially increase the human resources capacity for PMTCT in Tanzania.

## Abbreviations

ANC: Antenatal care
ART: Antiretroviral therapy
CHWs: Community health workers
DNA PCR: Deoxyribonucleic acid polymerase chain reaction
HIV: Human immunodeficiency virus
MoHCDGEC: Ministry of Health, Community Development, Gender, Elderly and Children
PEPFAR: President’s Emergency Plan for AIDS Relief
PLHIV: People living with HIV
PMTCT: Prevention of mother-to- child transmission of HIV
PNC: Postnatal care
UNAIDS: Joint United Nations Programme on HIV/AIDS
WHO: World Health Organization
